# Skin wound healing triggers epigenetic modifications of histone H4

**DOI:** 10.1186/s12967-020-02303-1

**Published:** 2020-03-26

**Authors:** Carlos H. V. Nascimento-Filho, Ericka J. D. Silveira, Eny M. Goloni-Bertollo, Lelia Batista de Souza, Cristiane H. Squarize, Rogerio M. Castilho

**Affiliations:** 1 Laboratory of Epithelial Biology, Department of Periodontics and Oral Medicine, University of Michigan, School of Dentistry, Ann Arbor, MI USA; 2 Genetics and Molecular Biology Research Unit, Department of Molecular Biology, School of Medicine of São José do Rio Preto, São José do Rio Preto, SP, Brazil; 3 Department of Oral Pathology, Federal University of Rio Grande do Norte, Natal, RN Brazil; 4 Michigan Medicine Rogel Cancer Center, University of Michigan, Ann Arbor, MI USA

**Keywords:** Histones acetylation, Repair and regeneration, Histone modification, H4K5, H4K8, H4K12, H4K16

## Abstract

**Background:**

The skin is the largest organ of the human body. Upon injury, the skin triggers a sequence of signaling pathways that induce epithelial proliferation, migration, and ultimately, the re-establishment of the epithelial barrier. Our study explores the unknown epigenetic regulations of wound healing from a histone perspective. Posttranslational modifications of histones enhance chromatin accessibility and modify gene transcription.

**Methods:**

Full-thickness wounds were made in the dorsal skin of twenty-four C57/B6 mice (C57BL/6J), followed by the use of ring-shaped silicone splints to prevent wound contraction. Tissue samples were collected at three time points (post-operatory day 1, 4, and 9), and processed for histology. Immunofluorescence was performed in all-time points using markers for histone H4 acetylation at lysines K5, K8, K12, and K16.

**Results:**

We found well-defined histone modifications associated with the stages of healing. Most exciting, we showed that the epidermis located at a distance from the wound demonstrated changes in histone acetylation, particularly the deacetylation of histone H4K5, H4K8, and H4K16, and hyperacetylation of H4K12. The epidermis adjacent to the wound revealed the deacetylation of H4K5 and H4K8 and hyperacetylation of H4K12. Conversely, the migratory epithelium (epithelial tongue) displayed significant acetylation of H4K5 and H4K12. The H4K5 and H4K8 were decreased in the newly formed epidermis, which continued to display high levels of H4K12 and H4K16.

**Conclusions:**

This study profiles the changes in histone H4 acetylation in response to injury. In addition to the epigenetic changes found in the healing tissue, these changes also took place in tissues adjacent and distant to the wound. Furthermore, not only deacetylation but also hyperacetylation occurred during tissue repair and regeneration.

## Background

The process of wound healing starts with the host response to an injury by inducing local inflammation, followed by the activation of cellular proliferation and tissue remodeling [1]. Each healing phase is governed by the activation and deactivation of genes [2], resulting from multiples signaling pathways and epigenetic modifications that fine-tune gene expression. Epigenetic modifications are constituted by a series of mechanisms that include histone acetylation, DNA methylation, and non-coding RNA, among others. Histones are the building blocks of the nucleosomes, working as spools that wind the DNA in an organized manner. Aside from the mechanical role, histones are also essential in gene regulation and overall chromatin organization. Histone modifications can be driven by acetylation, methylation, phosphorylation, ubiquitination, and sumoylation that constitute post-translation modifications. The complexity of histone modifications and the impact on the regulation of gene transcription is perceived as the “histone code” [3], and a better understanding of histone functions and modulations would pave the way to a better understanding and control of gene regulation in health and disease.

The acetylation of histones is considered a modification capable of changing the transcriptional state of the chromatin. Histone acetylation modulates transcription in two different ways, by a gain of function through the direct binding to chromatin-regulatory proteins like the bromodomains, or by undergoing a deacetylation process resulting in reduced transcriptional activity [4]. Histone H3 and H4 are the most highly conserved histones among different organisms and species. Of interest to this study, histone H4 contains four distinct acetylatable lysines (K5, K8, K12, and K16) with distinctive regulatory roles in gene transcription [5–7]. In yeasts, targeting individual lysine from histone H4 identified lysine 16 as the only acetylation site capable of inducing the expression of close to 100 unique genes not otherwise associated with lysines K5, K8, and K12 [8]. Mutations on lysines K5, K8, and K12 only resulted in a nonspecific change in gene expression [8].

Recent studies have started to profile the wound transcriptome [9–12]; however, changes in the epigenetic landscape of the epithelial barrier during healing is largely unknown. To this end, we used antibodies capable of identifying the specific acetylation status of histone H4 at lysines K5, K8, K12, and K16 during the process of healing. Our study identified specific histone modifications associated with different phases of wound healing. Our findings also identified surprising alterations on epigenetic markers located at a distance from the wound site indicating an unexpected and broader gene regulation of the epithelial barrier driven by an injury.

## Methods

### Experimental mice

Female and male mice C57BL/6J (Stock No: 000664, Jackson Laboratory, Bar Harbor, Maine) 4–6-week-old were housed in 12 h light/dark cycles. They received standard rodent chow and water ad libitum in compliance with the American Association for Accreditation of Laboratory Animal Care (AAALAC) guidelines. Mice were observed daily by the investigators and animal husbandry.

### Silicone splint and wound healing assay

Mice (n = 22) were anesthetized using a mixture of oxygen and isoflurane inhalation (Florane–Baxter Health Care Corporation). The dorsal skin was first shaved with an electric clipper, and the remaining hairs were removed with depilatory cream hair. Skin was rinsed with wet gauze, alcohol pads, and povidone-iodine topical solution. A full-thickness single wound was made on the back of mice with a 5 mm punch biopsy tool (extending to the panniculus carnosus). Surgical dye (India ink, Stat Lab, Lodi, CA) was applied to the wounded area to help with the subsequent clinical and histological localization of the wound, as described by Castilho et al. [13]. To minimize wound skin contraction in mice [14], we used ring-shaped silicone splints (cat.# GBLRD476687; Grace Biolabs, Bend, OR) centered and fixed to the skin nylon sutures as previously shown [15]. Wounds were allowed to heal by second intention. The wound size was monitored and measured daily with a digital caliper.

### Histological studies

Tissues were collected at post-operatory (post-op) days 1, 4, and 9 days (D1–D9). We excised the wound with the wound bed and margins, including approximately 5 mm of neighboring skin tissue. Next, the samples were fixed using 10% paraformaldehyde for 24 h (laid flat). The samples were bisected and further processed and embedded in paraffin. Histological Sections (5–8 microns) were deparaffinized in xylene substitute solution and rehydrated in a descending ethanol series. Histological analyses were performed on slides stained with Hematoxylin and Eosin (H&E). The borders and tissue zones were determined by an experienced pathologist. The *margin of the wound* was determined by the abrupt and clean cut of the epidermis and dermis and the presence of surgical dye. The *adjacent normal epidermis* is localized next to the wound and consists of 1–2 cell layers of keratinocytes recovering the connective dermis. Underneath the epidermis is a thin layer of muscle (panniculus carnosus), followed by the adventitia and underlying hypodermis (including fat). As the healing progresses, the *epithelial tongue* recovers the granulation tissue (dark basophilic tissue) that is present in the wound bed. The presence of hair follicles delimitates the edge of the adjacent normal epidermis from the wound bed or the epithelial tongue, depending on the healing phase. Lastly, the zone of *closed wound* is easily identified by the absence of hair follicles. At this point, the reepithelization is completed. The epidermis becomes a continuous thick layer with loss of rete pegs, adnexae, and hair follicles as previously described [16, 17].

### Immunofluorescence and quantification

Histological sections (5–8 microns) were used for the immunofluorescence reaction. Antigen retrieval was performed using citric acid buffer followed by blocking for unspecific binding using 3% (w/v) bovine serum albumin (BSA) in 0.5% (v/v) Triton X-100 phosphate-Buffered Saline (PBS). The tissue sections were incubated overnight with primary antibodies against Acetyl-Histone H4 (Lys 5) (PA5-40085, ThermoFisher); Acetyl-Histone H4 (Lys 8) (9HCLC, ThermoFisher); Acetyl-Histone H4 (Lys 12) (D2W6O, Cell Signaling Tech, Danvers, MA) Acetyl-Histone H4 (Lys 16) (E2B8W, Cell Signaling Tech.). Alexa Fluor 488 or 568 secondary antibodies (Invitrogen, Carlsbad, CA, USA) was used, followed by the Hoechst 33342 (blue stain-Invitrogen, Carlsbad, CA, USA) nuclear staining. Single tissue section was collected from each mouse to quantify each histone. Images were taken using a ×40 objective and a QImaging ExiAqua monochrome digital camera attached to a Nikon Eclipse 80i microscope (Nikon, Melville, NY, USA). Cell counting was performed on the images (1–3 fields) using the ImageJ^®^ software (National Institute of Mental Health, Bethesda, Maryland, USA). Only epidermis was included in the scoring. Labeling index or % of positive cells was defined as the number of positive cells (i.e., histone-Alexa Fluor 488 or 568 nuclear stain) divided by the total number of cells (blue nuclear stain) ×100.

### Statistical analysis

The statistical analyses were performed using GraphPad Prism 8 (GraphPad Software, San Diego, CA). A two-sided p-value of less than 0.05 (α = 0.05) was considered significant. Categorial variables (like the expression of histones at 2 sites) were analyzed using Welch’s t-test. For continuous variables, generally, one-way analyses of variance (ANOVA) were performed with Tukey’s post hoc tests for multiple comparisons. Kruskal–Wallis test (with Dunn’s post hoc tests) was used in lieu of ANOVAs due to the underlying correlativity of some variables. Data are expressed as mean ± standard error of the mean (SEM). Asterisks denote statistical significance (*p < 0.05, **p < 0.01, ***p < 0.001, ****p < 0.0001, and ns p > 0.05).

## Results

### Anatomical and histological analyses of the wound

**Fig. 1:**
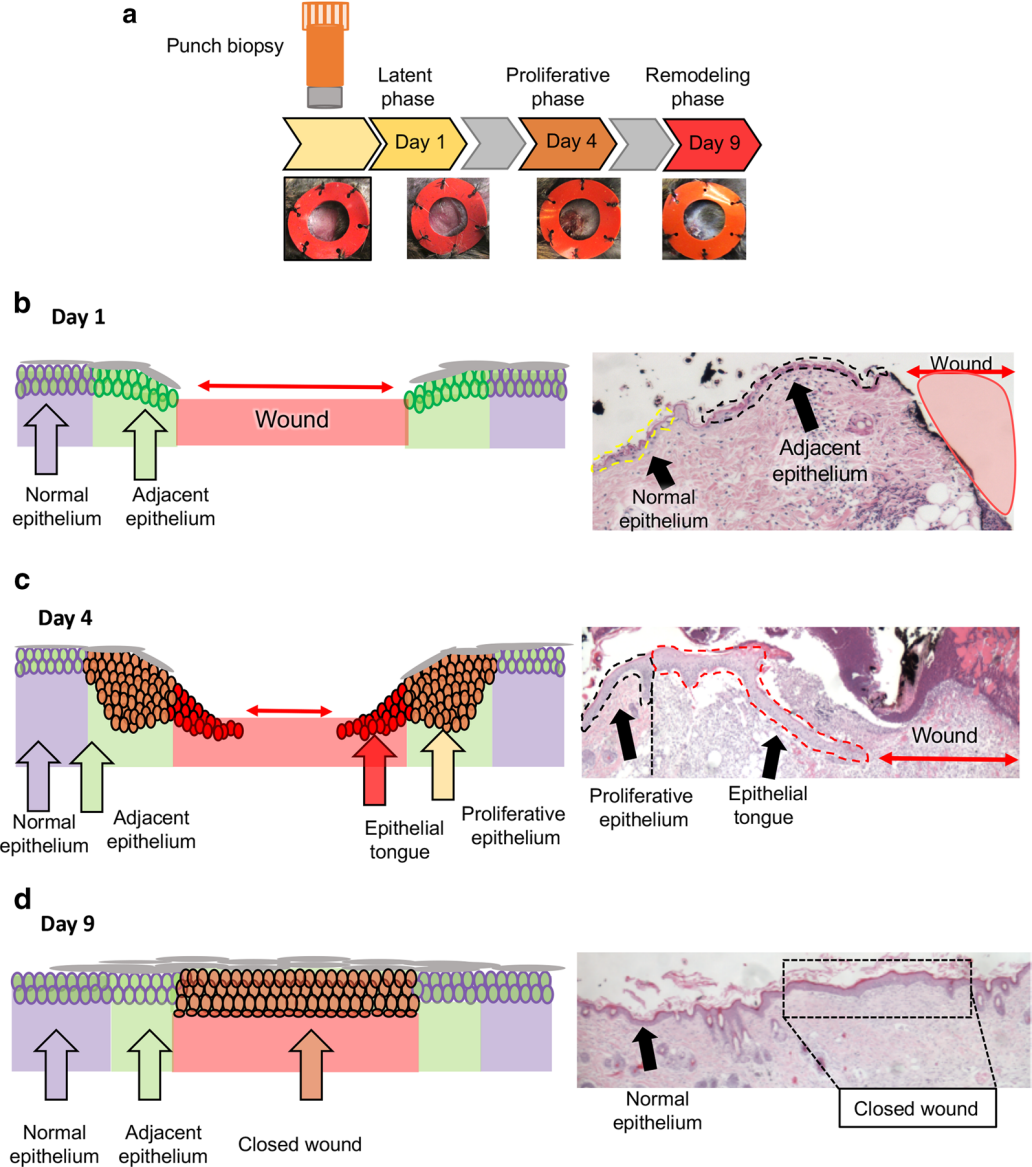
Epidermal wound and histological analysis: **a** Schematic representation of the experiment timeline showing the punch biopsy tool to create a 5 mm wound on the skin. Silicone splint was attached to the back of the mice to prevent wound contraction. Tissue samples were collected 1, 4, and 9 days after wounding (n = 8 mice per time point). **b** Schematic representation and H&E staining of wounds collected on day 1. Note the presence of thin adjacent epithelium next to the open wound of mice. **c** Schematic representation and H&E staining of wound collected on day 4. Note the formation of an epithelial tongue migrating underneath the wound scab, and the presence of a thick proliferative epithelium constituted by proliferative epithelial cells. **d** Schematic representation and H&E staining of wound area collected on day 9. The wound is closed (reepithelization) by day 9. The repaired epidermis presents a thick layer of epithelial cells that covers the wound (box) when compared to the normal epidermis (arrow)

The wound healing process can be divided into 3 phases, the latent (early phase), proliferative, and remodeling phases (Fig. 1a). The timeline starts with the injury day, and although the healing process is orchestrated by different types of cells that work in an organized fashion, it is the reepithelialization process that confers the protective barrier to a wound. Wound size directly affects the timeline of each wound phase. In our animal model, we divided the wound phase in post-injury day 1 that displays hemostasis and an initial latent period, showing a clear wound border (i.e., no epithelial migration) (Fig. 1b). Notably, we maintained a silicone splint in place throughout the process to minimize the effects of wound contraction commonly observed in mice. At post-op day 4, we observed distinct signs of reepithelialization, in which neoformed epidermis (i.e., epithelium tongue) migrates over the granulation tissue of the open wound (Fig. 1c). On post-op day 9, the reepithelization process was completed in our animal model (Fig. 1d).

### Tissue injury triggers broad deacetylation of histone H4 in epithelial cells

**Fig. 2:**
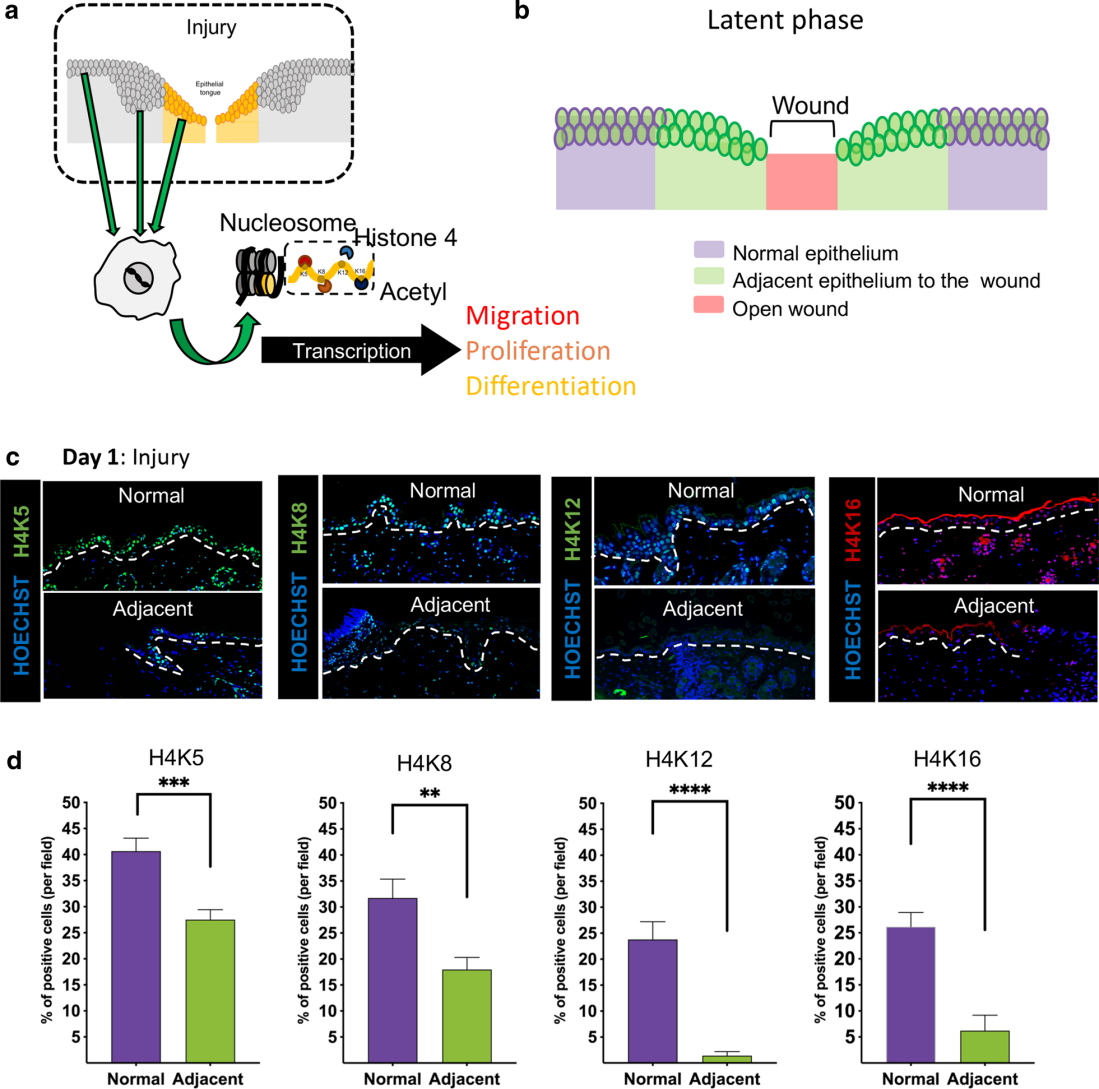
Tissue injury triggers broad deacetylation of histone H4 in epithelial cells. **a** Schematic representation of nucleosomes formed by histones with emphasis on histone H4 and its 4 acetylation sites. **b** Schematic representation of the latent phase of the wound, which is characterized by the absence of epithelial migration. **c** Representative images of immunofluorescence staining for histone H4 acetylated at lysine 5 (H4K5_green), lysine 8 (H4K8_green), lysine 12 (H4K12_green), and lysine 16 (H4K16_red), as well as Alexa 488 (green), Alexa 568 (red), and Hoechst 33342 (counterstain). Dashed line delineates the epidermis. **d** Quantification of positive epithelial cells (%) stained for acetyl histone H4 at lysines 5, 8, 12, and 16 (**p < 0.01, ***p < 0.001, ****p < 0.0001) (n = 8 mice; data represented as mean ± SEM)

Histone H4 is involved in the process of chromatin decompaction and gene transcription and plays an essential role in organismal response to environmental changes [18, 19]. Even though histone modifications play an essential role in fine-tuning gene transcription, the basic understanding of histone modifications in response to injury remains unknown (Fig. 2a). Here we decided to explore the impact of a cutaneous wound to the acetylation of histone H4 by exploring changes in the acetylation levels at the lysines K5, K8, K12, and K16. Initially, we characterized the latent phase of a wound (post-op day 1) (Fig. 2b) and observed a global deacetylation of the histone H4 at all lysines (Lys) at the margin of the wound site when compared to the observed at distant healthy tissues (day 1/latent phase) (Fig. 2c, d). Following, we sought to explore the acetylation pattern of histone H4 during migration and tissue healing.

### Histone H4 lys12 (H4K12) is acetylated during epithelial proliferation and migration

**Fig. 3:**
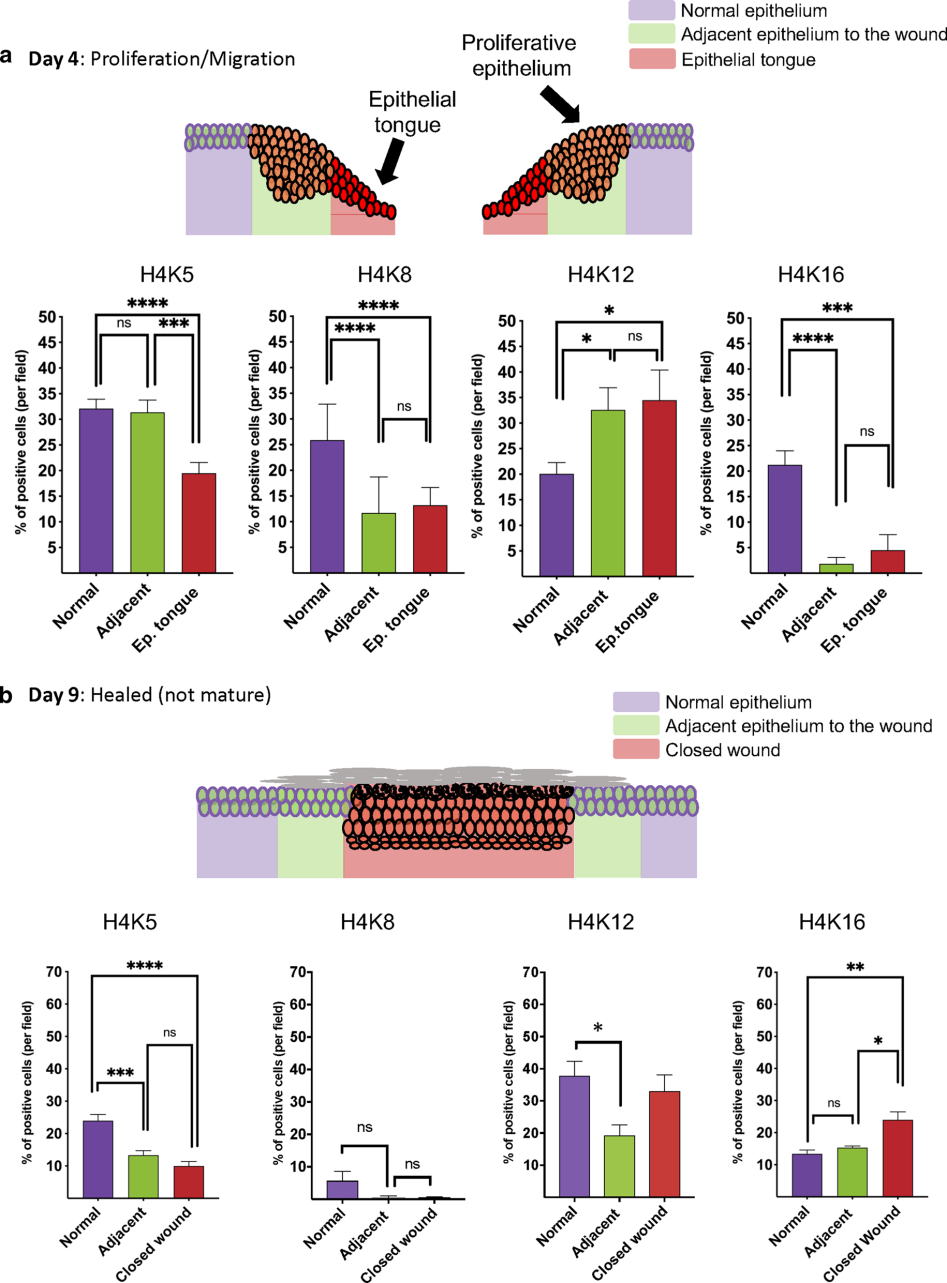
Changes in the acetylation pattern of lysines 5, 8, 12 and 16 during healing. **a** Schematic representation of the healing phase at day 4 shows the presence of normal epithelium, proliferative epithelium, and migratory epithelial tongue. Quantification of the percentage of epithelial cells positive for acetyl histone H4 at lysines 5, 8, 12, and 16 by day 4 after wound (n = 8 mice). Note deacetylation of histone H4 at lysines 5, 8, and 16, while lysine 12 is found hyperacetylated during the migration and proliferation phases (*p < 0.05, ***p < 0.001, ****p < 0.0001) (data represented as mean ± SEM). **b** Schematic representation of wound at day 9 depicts a complete wound closure. Quantification of the acetylation levels of histone H4 at lysines 5, 8, 12, and 16 shows overall deacetylation of lysines 5, 8, and 12, while lysine 16 is observed upregulated exclusively at the closed wound site (*p < 0.05, **p < 0.01, ***p < 0.001, ****p < 0.0001) (n = 6 mice; data represented as mean ± SEM)

During the process of wound healing, epithelial cells undergo a proliferation stage along with cellular migration evident as early as day 3 to 4 after the initial injury [20, 21]. Briefly, epithelial cells from the border of the wound start to proliferate (Fig. 3a), while epithelial cells next to the wound initiate a program of cellular migration extending over the wound bed as epithelial projections, also known as epithelial tongues. Interestingly, the acetylation levels of histone H4 on epithelial tongues remained downregulated when compared to healthy tissues except for H4K12. H4K12 shows increased acetylation levels at the adjacent epithelial cells and the epithelial tongue (Fig. 3a). It is also noted that histone H4 lys5 (H4K5) was the only histone presenting unchanged acetylation levels between normal and adjacent epidermis at post-injury day 4 (Fig. 3a).

### Histone H4 lysine 16 (H4K16) is enhanced in the closed wound area

Upon wound closure, epithelial cells stop migrating and proliferating due to contact inhibition. Next, the cells undergo a differentiation program that leads to epidermal stratification and consolidation of a new epithelial barrier [1]. The newly formed epidermis is characterized by an increased number of epithelial layers compared to the uninjured epithelium (Fig. 3b). We observed that our animal model for wound closure presents complete reepithelization after 9 days of the injury. When exploring the histone modifications that take place during healing, we noticed that the acetylation pattern of histone H4 dramatically changes upon wound closure. The most notable finding was that histone H4K16 was the only lysine found hyperacetylated at the healed epithelial area (Fig. 3b_H4K16).

### Closed wounds present a distinctive acetylation pattern of histones

**Fig. 4:**
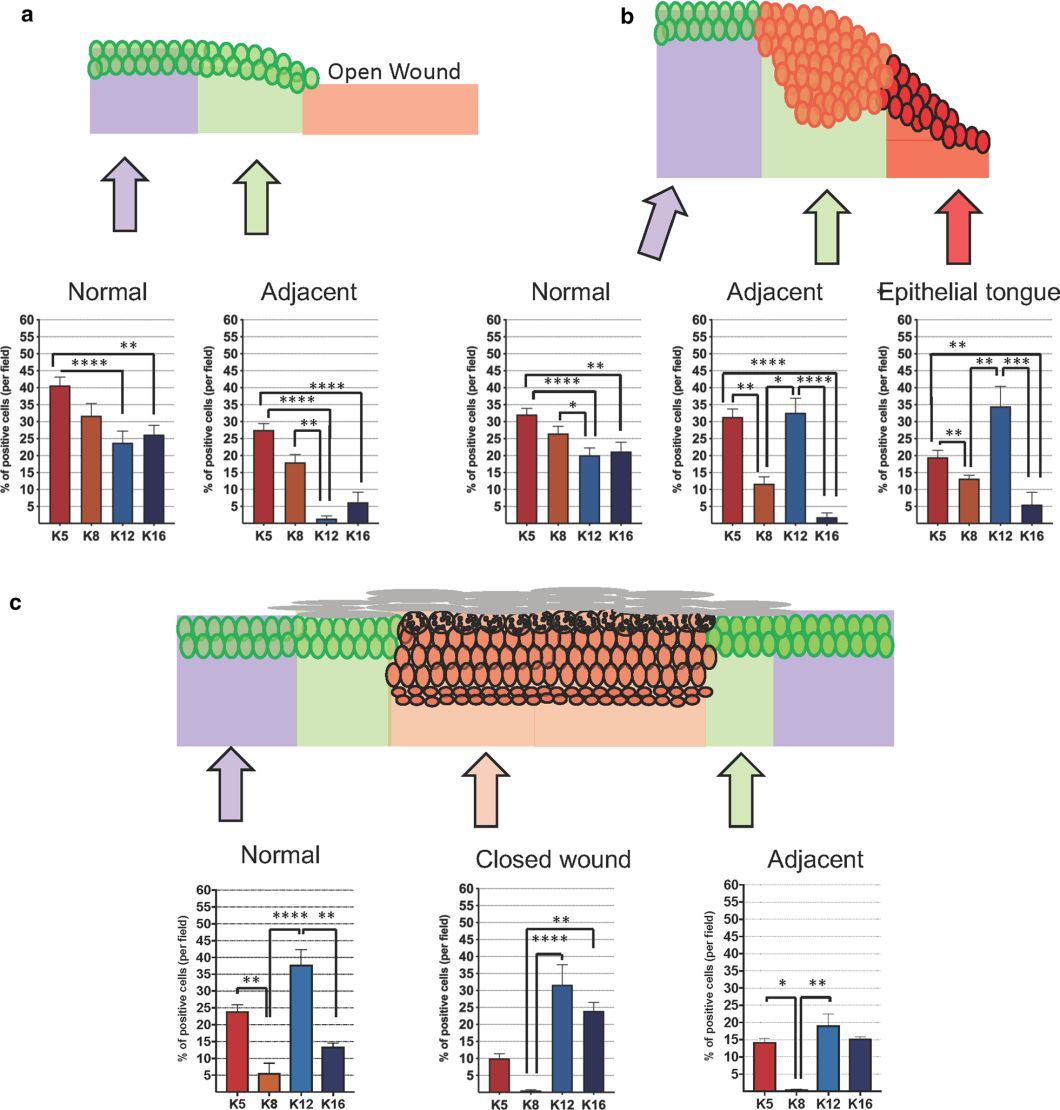
Changes in H4 acetylation pattern distributed among epithelial compartments. **a** Day 1 after wounding depicts the global deacetylation of histone H4 lysines at the adjacent and normal epithelia. Normal and adjacent epidermis display similar patterns (n = 8 mice). **b** H4K12 is hyperacetylated by day 4 after injury at the adjacent epithelium and epithelial tongue, compared to the normal epithelium (n = 8 mice). **c** On day 9, we observed the complete closure of the epidermis. H4K12 is shown hyperacetylated at the normal epidermis, and H4K16 is hyperacetylated at the closed wound site. Note that overall acetylation levels of H4K12 are higher than other acetylation sites of histone H4 (*p < 0.05, **p < 0.01, ***p < 0.001, ****p < 0.0001) (n = 6 mice; data represented as mean ± SEM)

Each phase of wound healing presents a distinct acetylation pattern of lysines with global deacetylation of histone H4 upon injury (Fig. 2c, d), while H4K12 is acetylated during migration (Fig. 3a), and H4K16 is acetylated upon complete epidermal closure of the wound bed (Fig. 3b). However, from a global perspective, we observed that the acetylation patterns of the histone H4 lysines were similar between normal and adjacent tissues on day 1 after injury, in which H4K5 and H4K8 had increased expression (Fig. 4a). On day 4, there was a significant increase expression of H4K12 at the adjacent epithelium and epithelial tongue (Fig. 4b). Healed tissues, however, presented complete deacetylation of histone H4K8 in normal, adjacent, and closed wound areas (Fig. 4c). It was also clear that among all acetylation sites of the histone H4, lysine 12 (K12) is the highest expressed in all anatomical areas at day 9 (Fig. 4c), whereas the acetylation K8 is downregulated in normal, adjacent, and closed wound areas after healing (Fig. 4c).

### Tissue injury induces distant changes in the acetylation pattern of histones over time

**Fig. 5:**
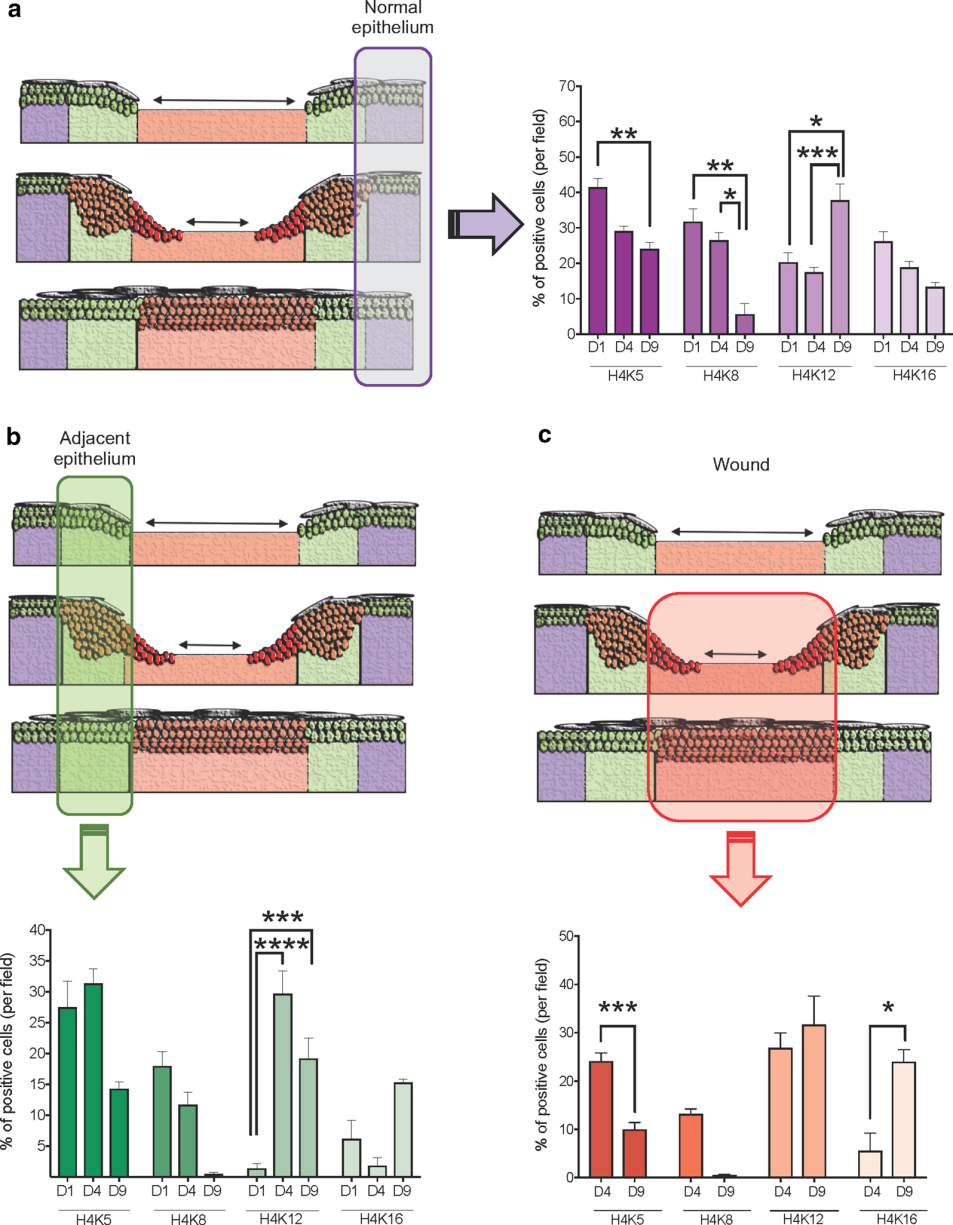
Changes in histone H4 acetylation observed in epidermal healing overtime. The three graphics show the time course for the change in the acetylation pattern of histone H4 at lysines 5, 8, 12, and 16 during the wound healing. **a** Note that the epidermis located distant from the wound presents continuous deacetylation of histone H4K5, H4K8, and H4K16, as the healing occurs. Hyperacetylation of H4K12 is found at the distant epithelium upon complete wound closure (reepithelialization at day 9). **b** Analysis of the epidermis adjacent to the wound revealed the deacetylation of H4K5 and H4K8, particularly upon wound closure (Day 9, reepithelialization). Conversely, significant hyperacetylation of H4K12 occurs at day 4 and day 9. **c** Changes in the epithelium recovering the wound were also observed. The migratory epithelial tongue displays significant deacetylation of H4K5 on day 9. The same expression pattern was observed for H4K8. The acetylation of H4K12 was unaltered upon wound closure; however, the epithelialized wound presented increased acetylation of H4K16. (*p < 0.05, **p < 0.01, ***p < 0.001, ****p < 0.0001) (data represented as mean ± SEM)

The process of wound healing mainly refers to the recovery of the wounded tissues achieved by the reestablishment of the epithelial barrier and long-term remodeling of the connective tissue. However, in the current study, we observed that the effects of an injury go beyond the wounded skin. We observed that the epidermis localized away from the wounded site responded to the trauma by changing the acetylation profile of histones (Fig. 5a). We observed that H4K5 underwent continuous deacetylation during healing. H4K12, however, became hyperacetylated at day 9. These findings indicate that tissue trauma triggers distant H4K12 acetylation and, consequently, gene transcription. Similar to the normal epithelium, the acetylation levels of histones H4K5 and H4K8 decreased during the healing of the skin (Fig. 5b, c). Interestingly, H4K12 became hyperacetylated at the adjacent epithelium and the wound reepithelialization (Fig. 5b, c). Epidermis covering the wound expressed hyperacetylation of H4K16 (Fig. 5c).

## Discussion

The human genome project was completed in 2003, covering 99% of the genome euchromatin and identifying close to 22,000 protein-coding genes [22]. Against initial predictions, the human genome is far smaller than anticipated suggesting that the maintenance of a complex multicellular organism requires additional regulatory machinery like histone modifications. Histones pack the chromatin in an organized way that allows unwinding of the DNA and fast gene transcription [23]. Histone modifications include acetylation, methylation, and phosphorylation. It is perceived that histone methylation results in stable modifications, while acetylation and phosphorylation of the histones can result in a transient modification [24]. Histone methylation is also often associated with reduced gene expression. For example, histone H3 lysine 27 trimethylation (H3K27me3) that has repressor activity is found downregulated during wound healing [25]. Along with the demethylation of H3K27me3, the process of cutaneous healing also displays the acetylation of H3K9 [26]. There is a limited number of studies that explore the role of histones during wound healing [25, 27]. Here, we decided to characterize the acetylation pattern of 4 different lysines from histone H4 during three distinct phases of healing.

We observed that histone acetylation does rapidly change its acetylation pattern during the different phases of healing. Perhaps one of the most unexpected and exciting results from this study is the modifications to the acetylation profile of histone H4 observed distant from the wound boundaries. Our findings point towards an epigenetic mechanism capable of modifying gene expression on the healthy area around the injury. Most exciting is that we have identified H4K12 as the primary lysine hyperacetylated at distant areas from the wound and potentially a marker to identify and characterize the distant effects of wounds. On the other hand, histone H4 lys5 (H4K5) maintained similar levels of acetylation between normal and adjacent tissues to the wound by day 4. This finding suggests that H4K5 is potentially associated with the suppression of genes transcription associated with differentiation, and consequent release of epithelial migration.

The acetylation of lys16 at histone H4 plays an important role in changing the conformation of the chromatin resulting in increased transcription though the reduced inter-nucleosome interaction and consequently increased chromatin accessibility to non-histone proteins [28]. We observed that histone H4K16 is the only lysine found hyperacetylated at the closed wound area. Interestingly that upon closure, the epidermis recovering the wound site undergoes a process of thickening suggestive of intense cellular differentiation, as observed in Fig. 1d. Cellular differentiation, as cellular senescence are essential protective mechanisms involved in reduced cellular proliferation and the maintenance of tissue homeostasis. In cancer, however, H4K16 is found hypoacetylated suggesting the disruption of cellular differentiation mechanisms and the potential in controlling tumor suppressor genes [29, 30]. Furthermore, the hypoacetylation of H4K16 is associated with dysfunctional DNA repair mechanisms in the animal model for Hutchinson Gilford progeria syndrome that result in the activations of an accelerated aging phenotype [31]. Combined, elevated genomic instability driven by the hypoacetylation of H4K16 can either trigger accelerated senescence in normal tissues or can lead to tumor progression driven by a dysfunctional DNA repair machinery.

Similar to H4K16, we have identified a spike on H4K12 acetylation during epithelial migration and proliferation at day 4 after injury. At the same time, all other lysines from histone H4 were deacetylated compared to healthy tissues. There is little information available on the role of lysine 12 acetylation of histone H4, especially in wound healing and epithelial biology. Acetylation of histone H4K12 along with H3K9 is associated with the maintenance of progenitor-specific characteristics of rod photoreceptor cells from the retina [32]. The maintenance of cycling progenitor cells was also associated with the inhibition of histone deacetylase 1 (HDAC1) that further prevented rod cells from undergoing differentiation. These results are somewhat aligned with our epidermal data in which migrating epithelial cells expressing high levels of H4K12 proliferate but do not undergo differentiation, as observed in the migratory epithelial tongue (Fig. 1d). From a cancer perspective, the hyperacetylation of histone H4K12 is associated with poor prognosis of pancreatic cancers [33] that, along with the rod cells data and our wound-healing results, may be correlated with the presence of undifferentiated tumor cells and reduced cellular differentiation.

## Conclusion

The mechanism involved in tissue repair upon an injury is comprised of a multi-step process that involves the mobilization of epithelial cells, the activation of an inflammatory response, and the remodeling of the wound bed. The wound-healing process is far from simple and is likely influenced by epigenetic modifications. Our current study points towards the presence of a histone code for epidermal repair, in which each healing phase presents a dominant histone H4 hyperacetylation site. These findings further facilitate the identification of epigenetic-controlled genes from each healing timepoint, and the testing of epigenetic drugs to treat skin wounds.

## Data Availability

Not applicable.

## Abbreviations

AAALAC: American Association for Accreditation of Laboratory Animal Care
IACUC: Institutional Animal Care and Use Committee
BSA: Bovine serum albumin
PBS: Phosphate-buffered saline
w/v: Weight per volume
SEM: Standard error of the mean
post-op: Post-operatory
H4K5: Histone H4 lysine 5
H4K8: Histone H4 lysine 8
H4K12: Histone H4 lysine 12
H4K16: Histone H4 lysine 16
H3K27me3: Histone H3 lysine 27 trimethylation
HDAC1: Histone deacetylase 1
H&E: Hematoxylin and eosin
